# Getah virus nonstructural protein 2 suppresses interferon-beta production by interrupting interferon regulatory factor 3 activation

**DOI:** 10.1186/s13567-025-01547-3

**Published:** 2025-06-07

**Authors:** Hua Liu, Zhao Qi, Lan Tian, Zhe Chen, Haonan Li, Le Liu, Sicong Liu, Shuai Li, Jiumeng Sun, Ying Shao, Xiangjun Song, Jian Tu, Liangqiang Zhu, Kezong Qi, Zhenyu Wang

**Affiliations:** 1 Anhui Province Key Laboratory of Veterinary Pathobiology and Disease Control, College of Veterinary Medicine, Anhui Agricultural University, Hefei, 230036 China; 2 Anhui Province Engineering Laboratory for Animal Food Quality and Bio-Safety, College of Veterinary Medicine, Anhui Agricultural University, Hefei, 230036 China; 3 Anhui Animal Disease Prevention and Control Center, Hefei, 230091 China; 4 Joint Research Center for Food Nutrition and Health of IHM, Anhui Agricultural University, Hefei, 230036 China

**Keywords:** Getah virus, nonstructural protein 2, IRF3, KPNA, type I interferon

## Abstract

Getah virus (GETV), a neglected and re-emerging mosquito-borne alphavirus, has become more serious and poses a potential threat to animal safety and public health. The innate immune response is critical for host defence against viral infection, and the dysregulation of host innate immune responses likely aggravates GETV infection. In this study, we use unbiased screening to identify GETV proteins that antagonise type I interferon (IFN-I) response. We found that GETV Nsp2 could inhibit Sendai virus or poly(I:C)-induced IFN-β promoter activation, potently suppressing primary interferon production— a key component of the host’s innate immunity antiviral response. Remarkably, Nsp2 showed efficient inhibition of the IRF3-responsive promoter, but not AP-1 or NF-κB. Further examination revealed that Nsp2 significantly suppressed luciferase activity when RIG-I-CARD, MDA5, MAVS, or IRF3 activated the IFN-β promoter. By contrast, IRF3/5D led to less suppression of luciferase expression, partially restoring luciferase activity, suggesting that Nsp2 interferes with the biological function of IRF3 as a crucial strategy in its antagonism of IFN-β production. Mechanistically, Nsp2 binds TBK1 to suppress IRF3 phosphorylation. Meanwhile, Nsp2 competitively inhibited the interaction of pIRF3 with KPNA3 and KPNA4, to inhibit IRF3 nuclear translocation. Overall, we demonstrated that GETV suppresses antiviral innate immunity by inhibiting the activation of IRF3, and Nsp2 plays a crucial role in this process. These findings reveal a novel strategy by which GETV evades the host innate immune response, providing new insights into the pathogenesis of GETV.

## Introduction

Getah virus is a mosquito-borne virus that belongs to the Semliki group of the *Alphavirus* genus within the family *Togaviridae*. It is a zoonotic arbovirus, meaning it can cause disease in both humans and animals [1].

First isolated from *Culex* mosquitoes in Malaysia in 1955, GETV has since been found in mosquitoes across various Asian countries surrounded by the Pacific Ocean (China, Japan, South Korea, Mongolia, Russia, and India), based on viral isolation and/or molecular epidemiological investigations [2].

GETV has a wide geographical distribution and a diverse host range. In livestock, infection primarily causes diseases in livestock such as pigs and horses, leading to fever, skin eruptions, and limb oedema in horses [3]. In pigs, GETV causes fever, anorexia, depression, diarrhoea, fetal death, and reproductive disorders [4, 5]. Recently, GETV has expanded its circulating territories due to the spread of Aedes mosquitoes caused by global warming [6]. GETV-neutralising antibodies have been detected in cattle and humans, suggesting a potential public health risk and posing an increasing threat to animal industries [6–8].

GETV, like other typical alphaviruses, is an enveloped virus with a positive-strand RNA genome measuring 11.7 kb. The genome includes a 5′-untranslated region (UTR), two open reading frames (ORFs), a 3′-UTR, and a poly-A tail (19). The first ORF, located at the 5′ end, encodes nonstructural proteins Nsp1, Nsp2, Nsp3, and Nsp4. The second ORF, found at the 3′ end, encodes the structural proteins capsid (C), E3, E2, 6 K, and E1 [9].

The virions are spherical particles approximately 70 nm in diameter and consist of three layers: the inner viral core, the capsid, and the outer glycoprotein-decorated envelope [10, 11]. The E1 and E2 proteins form heterodimers that are immobilised in the membrane. The structural proteins encapsulate the viral nucleic acids and facilitate the assembly of viral particles. The nonstructural polyprotein is translated directly from the genomic RNA (gmRNA), while the structural polyprotein is translated from a subgenomic mRNA (sgmRNA), which is transcribed by the viral replicase from an internal promoter [12].

The nonstructural proteins play essential roles in viral replication, translation, and the evasion of the host immune response. Therefore, both structural and nonstructural proteins are considered promising targets for the development of antiviral drugs against GETV [4].

The innate interferon (IFN) response is one of the primary defences that the host uses against viral infections. IFNs are highly effective in limiting both the replication and spread of viruses. Since these proteins are typically not produced in the absence of a viral threat, the synthesis of IFNs synthesis must be triggered quickly and strongly when the host encounters a virus.

The IFN-mediated antiviral pathway consists of two main stages: IFN activation and IFN signalling [13]. When infection occurs, viral pathogen-associated molecular patterns (PAMPs) are detected by host pattern recognition receptors (PRRs) [14, 15]. This recognition activates host protein signalling cascades, leading to the activation of transcription factors such as interferon regulatory factor 3 (IRF3) and NF-кB. The collaboration of these factors results in the expression of type I IFNs [16–18].

Once secreted, IFN-α/β act as autocrine and paracrine factors, inducing the expression of IFN-stimulated genes (ISGs). This process triggers the expression of hundreds of ISGs that possess antiviral functions, resulting in a significant antiviral state in the host [16, 19–21].

To successfully infect and replicate within their hosts, viruses have developed powerful strategies to counteract host innate immune activation. Research has shown that alphaviruses employ various methods to evade the host immune response, including antagonising IFN production, inhibiting IFN signalling, and enhancing IFN resistance, such as CHIKV, SINV, VEEV [22–24].

Currently, the mechanisms by which GETV regulates the innate immune response and its role in pathogenesis remain unclear. This study examines the interaction between GETV and host antiviral responses, with a focus on individual GETV proteins as potential suppressors of IFN-I production. These suppressors were mapped to identify their inhibitory effects in the IFN-I production pathways.

Our findings reveal that the GETV Nsp2 protein diminishes the ability of cells to produce IFN-β by interfering with the activation of IRF3. Nsp2 effectively reduces the phosphorylation of IRF3 and binds to nuclear transport proteins, specifically karyopherin α3 (KPNA3, also known as importin α4) and karyopherin α4 (KPNA4, also known as importin α3). This binding competitively disrupts the interaction between KPNA3, KPNA4, and IRF3, thereby inhibiting the nuclear translocation of IRF3.

These findings suggest a novel strategy by which GETV undermines cellular innate immunity and evades host antiviral responses.

## Materials and methods

### Cells, plasmids and viruses

The permanent PK15 cell line was obtained from BOSTER Bioengineering Co., LTD (Wuhan, China). The HEK293 T cells, Sendai virus, the pGL4 basic vector and the pRL Renilla luciferase plasmids were donated by Professor Yong Huang’s laboratory at the Engineering Research Center of Efficient New Vaccines for Animals, Ministry of Education, China [25].

Gene sequences for porcine MDA5, MAVS, TBK1, IRF3, KPNA3, KPNA4, and the IFN-β promoter, which contains 348 bp (from the sequence −297 to + 51), were sourced from the National Center for Biotechnology Information (NCBI) gene bank. The transcriptional binding regions PRDIV, PRDIII/I, and PRDII are recognised by the AP-1 family proteins, IRF family proteins, and NF-κB transcription factor, respectively [26, 27].

The AP-1/NF-κB-Luc reporter plasmid was created by deleting the PRDIII/I region from the IFN-β reporter plasmid. In contrast, the IRF3-Luc reporter plasmid was produced by ligating four copies of the PRDIII/I region. The nine genes of the Getah virus (GenBank: MF741771.1) were cloned into the pCAGGS-HA vector. Plasmid DNA was used to transform E. coli Dh5alpha, and the resistance of the transformants was determined.

The cells used in the experiments were cultured in Dulbecco’s modified Eagle’s medium (DMEM) (12100-046; Invitrogen Carlsbad, CA, USA), supplemented with 10% heat-inactivated Fetal Bovine Serum (Prime) (FSP500; ExCell Bio, Suzhou, China) and penicillin/streptomycin (100 U/mL). These cultures were maintained at 37 °C in a humidified atmosphere with 5% CO_2_, and all cells were tested to ensure they were mycoplasma-free.

### Antibodies and reagents

The following antibodies were used in this study: TBK1 Monoclonal antibody (Cat No. 67211-1-Ig), Phospho-TBK1 (Ser172) Recombinant antibody (Cat No. 82383-1-RR), IRF3 Monoclonal antibody (Cat No. 66670-1-Ig), Phospho-IRF3 (Ser396) Polyclonal antibody (Cat No. 29528-1-AP), all purchased from Proteintech. Additional reagents included mouse anti-HA antibody (H3663; Merck Sigma Aldrich), rabbit anti-histone H3 monoclonal antibody (BM4389; Wuhan Boster Biotech), Mouse anti-β-actin antibody (A00702; GenScript Biotech Corporation), mouse anti-Flag antibody (AE005; ABclone), and poly(I:C) (tlrl-pic) from InvivoGen. Protein G-agarose (sc-2002) and protein A-agarose (sc-2001) were obtained from Santa Cruz. DAPI (C1005) was sourced from Beyotime Biotechnology.

### Luciferase reporter assay

Cells were cultured until they reached 80% confluency. The promoter sequence containing the firefly luciferase reporter was amplified and cloned into the pGL4 basic vector (Promega). The cells were then transfected with the firefly luciferase reporter plasmid along with the normalising control vector pRL Renilla luciferase (Promega) using Lipofectamine 2000 (11668-019; Invitrogen). Following this, the cells were transfected with Nsp2 or another plasmid expressing RIG-I-CARD (a constitutively active form of RIG-I and a well-established inducer of IFN production), MDA5, MAVS, IRF3, IRF3/5D (a phosphor-mimic of the activated IRF3), KPNA3 or KPNA4. The cells were subsequently infected with Sendai virus or stimulated with poly(I:C). Luciferase activities were measured using the Dual-Luciferase Reporter System, and relative firefly luciferase activity was calculated as the ratio of firefly luminescence to Renilla luminescence.

### Co-immunoprecipitation and western blotting

Cells cultured in 100-mm-diameter dishes (Thermo Fisher) were transfected with the specified plasmids using Lipofectamine 2000. After 36 h, the cells were lysed on ice for 30 min with a lysis buffer composed of 150 mM NaCl, 50 mM Tris–HCl (pH 7.4), 1% Nonidet P-40, 0.5% Triton X-100, 1 mM EDTA, 0.1% sodium deoxycholate, 1 mM dithiothreitol, 0.2 mM phenylmethylsulphonyl fluoride, and a protease inhibitor cocktail [Sigma-Aldrich]). The cell lysate supernatant was collected by centrifugation and precleared by incubating with protein G/protein A agarose for 1 h at 4 °C.

Next, the supernatant was incubated overnight at 4 °C with the indicated antibodies and then precipitated with protein G agarose/protein A agarose for 30 min at room temperature. The precipitated complexes were centrifuged at 2000 × *g* for 10 s and washed three times with PBS. Finally, the bound proteins were eluted by boiling for 10 min in 2 × loading buffer, followed by SDS-PAGE and immunoblotting. Immunoreactive bands were visualised using enhanced chemiluminescence (ECL) reagents (Bio-Rad).

### Confocal microscopy

Cells grown on coverslips in 24-well culture plates were either transfected with the indicated plasmids or infected with the virus for the specified duration. Following this, the cells were fixed with 4% paraformaldehyde for 20 min at room temperature and permeabilised with 0.1% Triton X-100 for 15 min at room temperature. After washing with 0.1 M phosphate-buffered saline (PBS), the cells were preincubated with 2% bovine serum albumin for 1 h at 37 °C.

Next, the cells were incubated overnight with primary antibodies and subsequently with secondary antibodies for 1 h at 37 °C, followed by three washes as described above. After a brief staining with DAPI, the coverslips were mounted onto glass slides using a fluorescence mounting medium. All samples were analysed using a Leica STELLARIS 5 laser scanning microscope.

### Statistical analysis

Statistical analyses were performed using GraphPad Prism software version 8.0.1. Data are represented as mean ± standard error of the mean (SEM) (standard deviation [SD]). Differences between the experimental and control groups were assessed using one-way ANOVA, accompanied by Levene’s test. The Benjamini–Hochberg procedure was implemented to control the false discovery rate. Statistically significant and highly significant results were defined as *P* < 0.05 and *P* < 0.01, respectively.

## Results

### Getah virus proteins interfere with IFN-I activation

**Figure 1:**
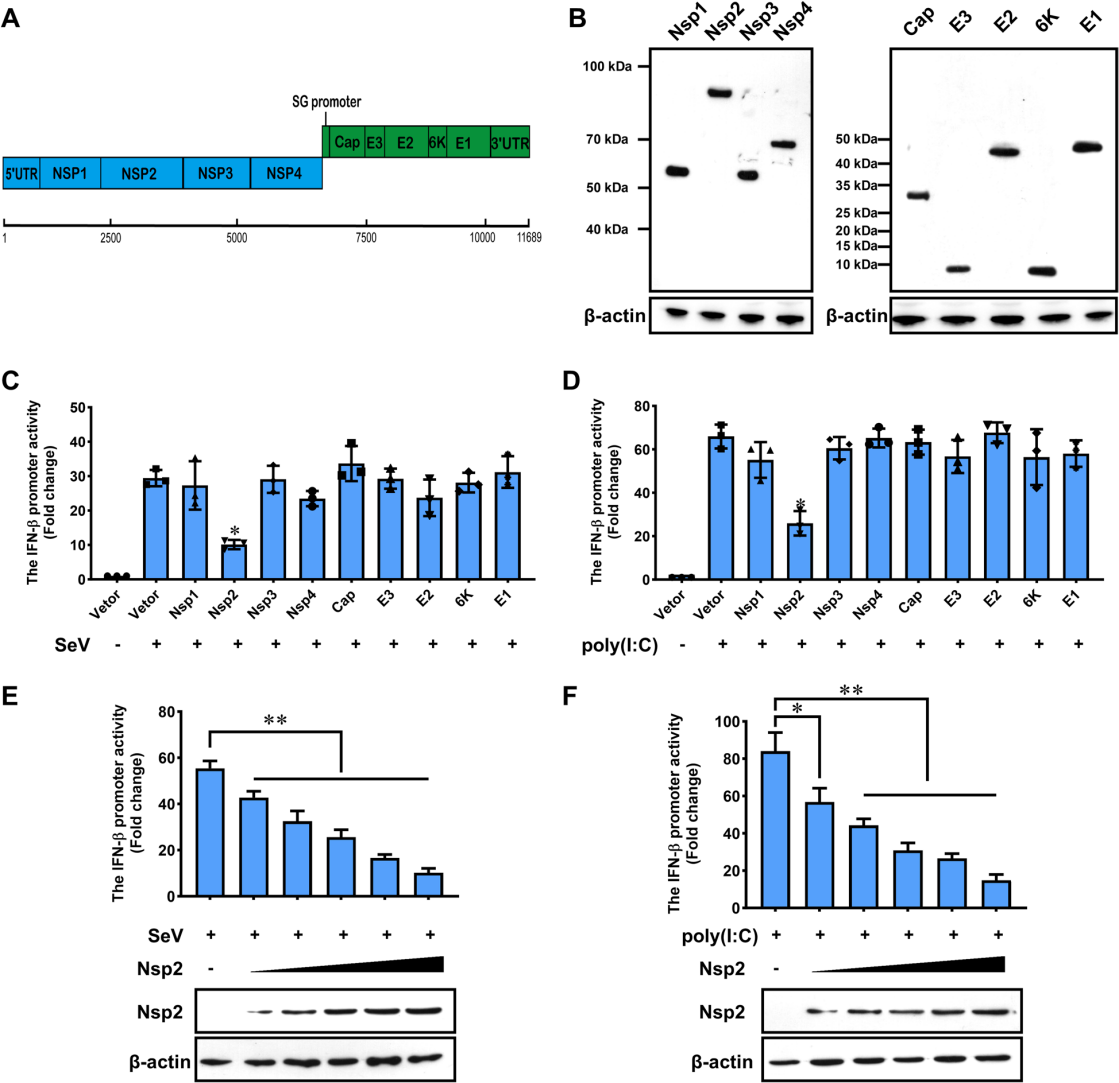
**Getah virus proteins interfere with IFN-I activation**. **A** Genome structure of Getah virus. **B** Expression of Getah virus proteins. The respective viral sequences were cloned into pCAGGS-HA vector and expressed in HEK293 T cells, subsequently analysed by western blotting using an anti-HA antibody. **C**, **D** HEK293 T cells were transfected with an IFN-β reporter plasmid, along with a control plasmid or with plasmids expressing the indicated GETV proteins. At 24 h post-transfection, cells were infected with SeV for 12 h or stimulated with poly(I:C) (200 ng) for 12 h, then the luciferase activity was measured. All experiments were performed at least twice, and a representative result is shown. Error bars indicate the SD of technical triplicates. One-way ANOVA determined statistics with Levene’s test, and the Benjamini–Hochberg procedure was applied to control the false discovery rate. Statistical significance was determined as follows: * *P* < 0.05. **E**, **F** HEK293 T cells were transfected with an IFN-β reporter plasmid, along with a control plasmid or with increasing amounts of plasmids expressing Nsp2 (5 ng, 50 ng, 100 ng, 200 ng, 300 ng). Cells were infected with SeV for 12 h or stimulated with poly(I:C) (200 ng) for 12 h and assayed for luciferase activity. The statistical significance of the presented data was determined as follows: * *P* < 0.05, ** *P* < 0.01.

Getah virus encodes nine viral proteins, including four non-structural proteins and five structural proteins (Figure 1A). To characterise the interferon antagonism of this neglected and re-emerging virus, we constructed a complete panel of expression plasmids, pcDNA3.1–3 × Flag, that encode the nine individual GETV viral proteins after codon optimisation for interferon antagonist screening. Western blot analysis revealed that all genes could be expressed, although at varying levels (Figure 1B).

To screen for GETV proteins that could inhibit IFN-β production, we transiently transfected 293 T cells with the vector plasmid or with plasmids expressing GETV proteins. We also induced a plasmid encoding a luciferase gene driven by the IFN-β promoter (pIFN-β-luc) and a control pRL Renilla luciferase for normalising transfection efficiency. After 24 h, the cells were stimulated with Sendai virus for 12 h, and we measured luciferase activity.

Our findings revealed that GETV proteins exhibited divergent effects on Sendai virus-induced IFN-β promoter activation. Sendai virus significantly increased IFN-β-luc reporter activity compared to mock-infected cells, indicating that the virus enhances IFN-β production. However, in cells transfected with Nsp2, the IFN-β-Luc reporter activity was significantly inhibited following Sendai virus infection (Figure 1C). Similar results were observed when cells were transfected with Nsp2 for 24 h then stimulated with poly(I:C) for 12 h (Figure 1D).

Furthermore, we confirmed that Nsp2 inhibits the activation of the IFN-β promoter in a dose-dependent manner in response to Sendai virus and poly(I:C), which are thought to stimulate the RIG-I and MDA5 signalling pathways, respectively (Figures 1E, F). These results suggest that GETV Nsp2 may play a crucial role in regulating the host's innate immune response.

### Getah virus Nsp2 inhibited IFN-β expression through IRF3 rather than through transcription of AP-1 or NF-κB

Activation of the IFN-β promoter needs Interferon Regulatory Factor 3 (IRF3), along with Nuclear Factor kappa-light-chain-enhancer of activated B cells (NF-κB) and Activator Protein 1 (AP-1). These factors bind to PRD III/I, PRD II, and PRD IV in the promoter sequence, respectively [28].

To identify the transcription factors involved in the inhibition of IFN-β production upon transfection with GETV Nsp2, PK-15 cells were co-transfected with the pRL-TK plasmid and the AP-1&NF-κB-Luc reporter plasmid or the IRF3-Luc reporter plasmid. The activity of the AP-1&NF-κB Luc and IRF3-Luc reporters was then assessed using a Dual-Luciferase Reporter (DLR) assay.

**Figure 2:**
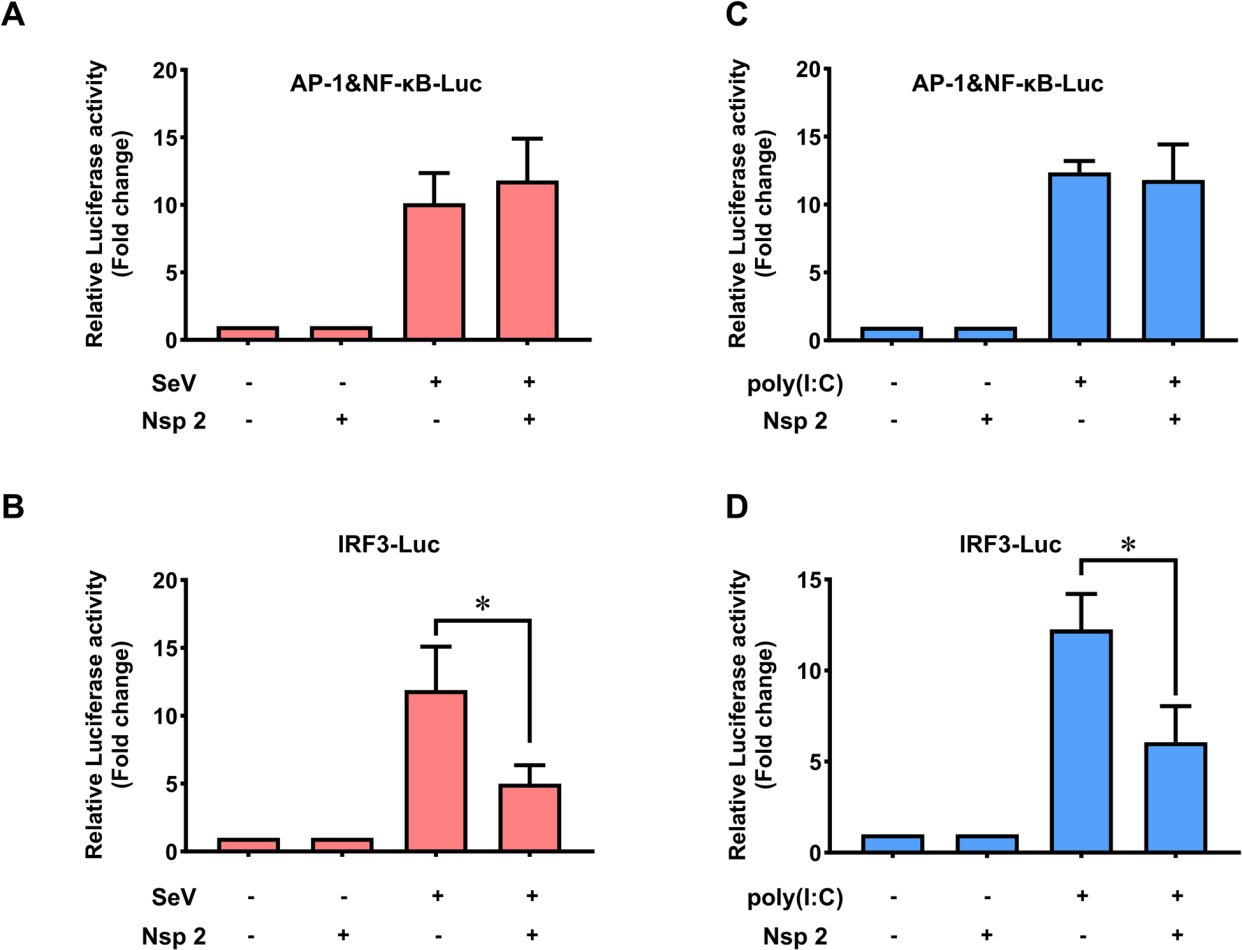
**Inhibition of the activity of transcription factors on specific PRDs of the IFN‐β promoter by GETV Nsp2.** PK-15 cells were transfected with Nsp2 (200 ng) for 12 h before being co-transfected with pRL-TK plasmid (50 ng) and AP-1&NF-κB-Luc (50 ng) or IRF3-Luc (50 ng) reporter plasmids. 24 h later, cells were infected or stimulated with or without SeV (**A**, **B**) or poly (I:C) (**C**, **D**). Luciferase activity was measured. Statistical significance of the presented data was determined as follows: * *P* < 0.05.

The results showed that Sendai virus infection and poly(I:C) transfection increased the activity of both the IRF3-Luc and AP-1&NF-κB-Luc reporters in cells transfected with the vector control (Figure 2). However, transfection with Nsp2 significantly diminished the increase in IRF3-Luc activity induced by either Sendai virus or poly (I:C). In contrast, the activity of the AP-1&NF-κB-Luc reporter remained unchanged. This indicates that the inhibition of IFN-β expression caused by Nsp2 is related to the IRF3 signalling pathway rather than the NF-κB and AP-1 signalling pathways (Figure 2).

### Getah virus Nsp2 suppresses IRF3 activation

Based on the results mentioned above, we confirm that GETV Nsp2 can effectively inhibit the activation of the RIG-I and MDA5 signalling pathways. To determine which steps of the RIG-I signalling cascade are blocked by GETV Nsp2, we co-transfected Nsp2 expression plasmids with the IFN-β promoter plasmid, in addition to a plasmid expressing RIG-I-CARD (a constitutively active form of RIG-I and a well-established inducer of IFN production), MDA5, MAVS, IRF3 or IRF3/5D (a phosphor-mimic of the activated IRF3). This setup was used to activate specific steps of the RIG-I pathway, after which we assessed the activation of the IFN-β promoter.

**Figure 3:**
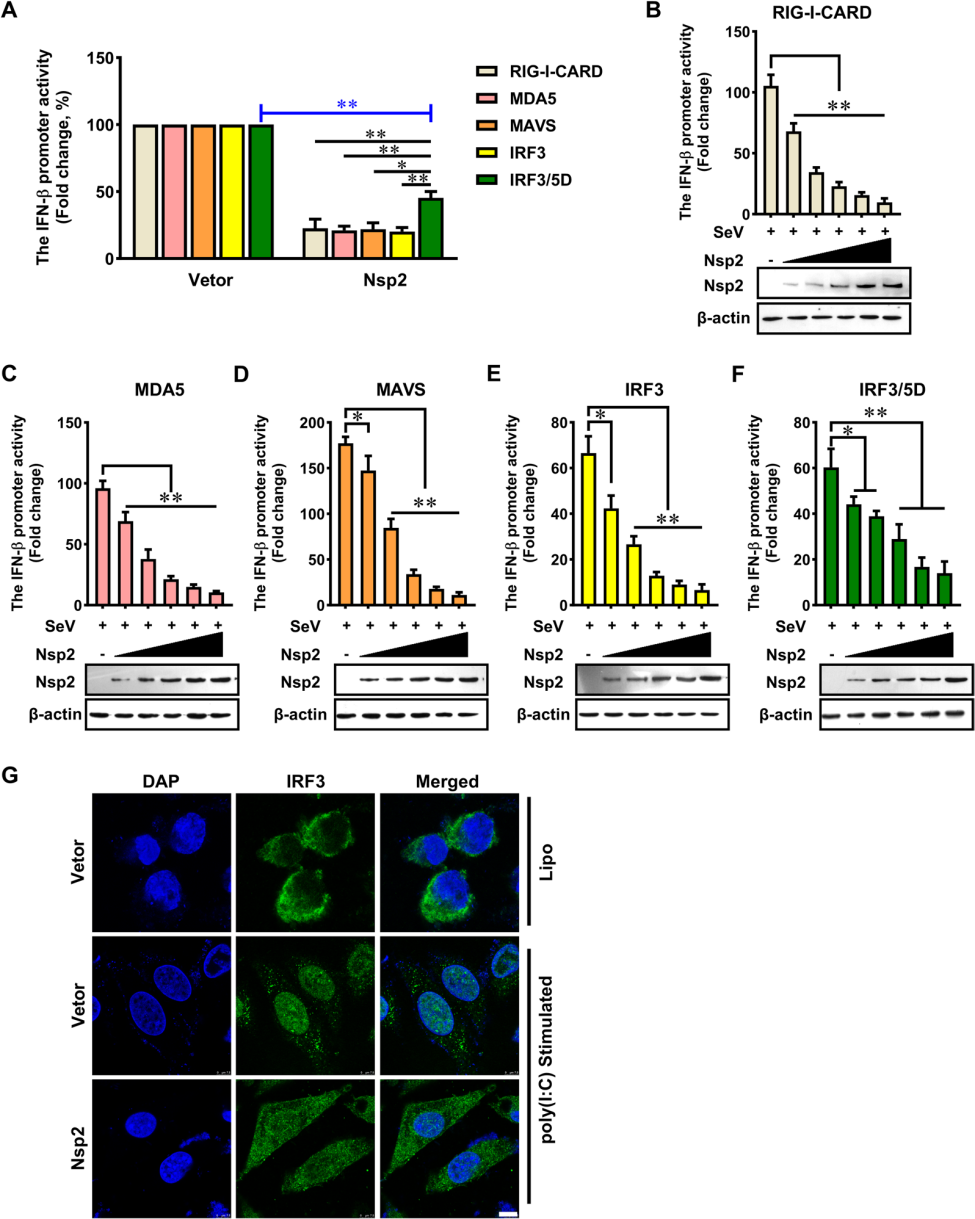
**Getah virus Nsp2 suppresses IRF3 activation**. **A** HEK293 T cells were co-transfected with IFN-β reporter plasmid, viral protein expressing plasmid, and stimulator plasmid RIG-I-CARD, MDA5, MAVS, IRF3 and IRF3/5D. An empty plasmid was used as a control. Cells were assayed for luciferase activity at 24 h. The data were analysed by normalising the Firefly luciferase activity to the Renilla luciferase activity and then normalising it to non-stimulated samples to obtain the fold induction. Empty vector control was set to 100%. **B**–**F** HEK293 T cells were transfected with an IFN-β reporter plasmid, along with a control plasmid or with an increasing amount of plasmids expressing Nsp2, together with plasmids expressing RIG-I-CARD, MDA5, MAVS, IRF3 and IRF3/5D. At 24 h post-transfection, cells were infected with SeV for 12 h, then the luciferase activity was measured. The statistical significance of the presented data was determined as follows: * *P* < 0.05, ** *P* < 0.01. **G.** PK-15 cells were transfected with the Nsp2-expressing plasmid. After 24 h, cells were treated with poly(I:C) for 12 h. The cells were fixed and subjected to laser scanning confocal microscopy. Scale bar, 10 μm.

The results demonstrated that GETV Nsp2 significantly suppressed luciferase activity when the IFN-β promoter was activated by RIG-I-CARD, MDA5, MAVS, or IRF3 (Figure 3A). In contrast, IRF3/5D partially restored luciferase activity (approximately, from 20 to 45%), but this restoration was not sufficient to reach the full luciferase activity observed in the vector control group (Figure 3A). Additionally, the overexpression of Nsp2 inhibited RIG-I-CARD, MDA5, MAVS, IRF3 and IRF3/5D-triggered IFN-β promoter activation in a dose-dependent manner (Figures 3B–F).

These findings suggest that Nsp2 may antagonise IFN-β production through multiple targets, possibly by affecting IRF3 (before IRF3 activation) or by targeting another component upstream of IRF3 (between TBK1 and IRF3), or even a downstream component of IRF3.

Subsequently, we examined the effect of Nsp2 on IRF3 nuclear translocation. Immunofluorescence analyses revealed that IRF3 translocated to the cell nucleus in the absence of Nsp2 following poly(I:C) treatment, whereas the expression of Nsp2 blocked its nuclear translocation (Figure 3G). Collectively, these findings indicate that GETV Nsp2 acts as an antagonist to IFN-β production primarily by diminishing IRF3 activation.

### Getah virus Nsp2 combines with TBK1 to decrease IRF3 phosphorylation

IRF3 phosphorylation and nuclear translocation are essential steps for the activation of IFN-β. Both RIG-I and MDA5 trigger antiviral signalling pathways through MAVS. The activation of MAVS leads to the stimulation of the kinases TBK1 and IKK. TBK1 subsequently phosphorylates IRF3, promoting its dimerisation, its translocation to the nucleus, and its regulation of de novo gene expression.

**Figure 4:**
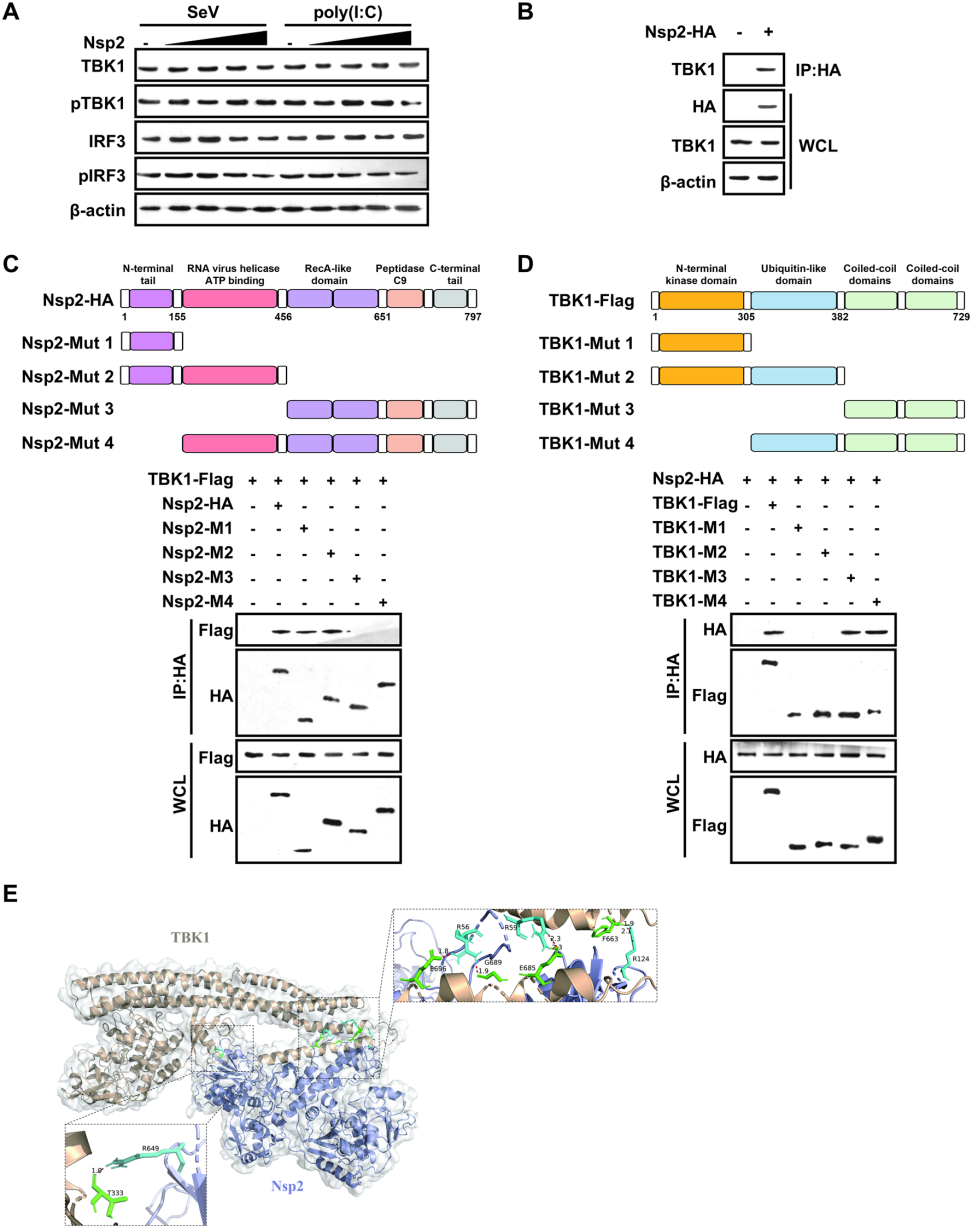
**Getah virus Nsp2, combined with TBK1, decreases IRF3 phosphorylation**. **A** PK-15 cells were co-transfected with TBK1-expressing plasmid and varying amounts of Nsp2-encoding plasmids. After 24 h, western blot was used to analyse the cell lysates for phosphorylated TBK1, total TBK1, phosphorylated IRF3, total IRF3, and β-actin. **B.**HEK293 T cells were co-transfected with plasmids expressing TBK1 and HA-tagged Nsp2. After 24 h, cell lysates and immunoprecipitates were immunoblotted with the indicated antibodies. **C.** Schematic representation of GETV Nsp2 domains according to its amino acid sequence. HEK-293 T cells were transfected with HA-tagged Nsp2 truncated fragments and Flag-TBK1 for 24 h. Cell lysates and immunoprecipitates were subjected to immunoblotting with the indicated antibodies. **D** Schematic representation of TBK1 domain positions. HEK-293 T cells were transfected with Flag-tagged TBK1 truncated fragments and HA-Nsp2, and co-immunoprecipitation and immunoblot analysis were performed after 24 h. **E** Docked complex of Nsp2-TBK1. Wheat shows the TBK1, and light blue represents the Nsp2; TBK1-binding residues are shown in green, and Nsp2-binding residues are displayed in cyan. Red represents the hydrogen bonds.

Given the critical role of TBK1 in phosphorylating and activating IRF3, we initially focused on the regulatory relationship between GETV Nsp2 and TBK1. We co-transfected PK-15 cells with a plasmid expressing TBK1 and Nsp2 and analysed the cells using western blotting. The results indicated that the expression of Nsp2 did not impact the levels of TBK1 protein induced by Sendai virus infection or poly(I:C) stimulation (Figure 4A). Interestingly, we found that Nsp2 did not inhibit TBK1 phosphorylation in a dose-dependent manner. However, Nsp2 did suppress IRF3 phosphorylation in a dose-dependent fashion when cells were subjected to Sendai virus infection or poly(I:C) stimulation (Figure 4A).

To investigate whether Nsp2 interacts with TBK1, we performed co-immunoprecipitation, revealing that Nsp2 could pull down TBK1 (Figure 4B). To identify the specific domain(s) of Nsp2 or TBK1 involved in their interaction, we created truncation mutants of both proteins. The analysis demonstrated that the interaction between Nsp2 and TBK1 depended on the N-terminus of Nsp2 and the two coiled-coil (CC) domains of TBK1 (Figures 4C and D).

To clarify the molecular connections between Nsp2 and TBK1, we conducted rigid-body docking using the ClusPro 2.0 web server. The calculated solvation-free energy gain upon forming the interface (ΔiG) for the Nsp2 and TBK1 binding complex was calculated using PDBePISA to be −19.0 kcal/mol (Figure 4E). Docking analysis revealed the formation of hydrogen bonds and salt bridges between the N-terminus of Nsp2 and the CCD2 of TBK1, with average bond lengths of 2.01 Å and 3.0 Å, respectively, consistent with our co-immunoprecipitation results. A detailed list of amino acid residues potentially involved in this binding is provided in Table 1.

**Table 1:** **Hydrogen bonds and salt bridges connected by Nsp2 and TBK1**

No	Type	NSP2	Dist. [Å]	TBK1
1	Hydrogen bonds	B: ARG 649 [HH21]	1.92	A: THR 333 [O]
2		B: ARG 124 [HH11]	2.06	A: PHE 663 [O]
3		B: ARG 124 [HH21]	1.91	A: PHE 663 [O]
4		B: ARG 59 [HH22]	2.30	A: GLU 685 [OE1]
5		B: ARG 59 [HE]	2.26	A: GLU 685 [OE1]
6		B: ARG 56 [HH21]	1.88	A: GLY 689 [O]
7		B: ARG 56 [HH12]	1.79	A: GLU 696 [OE2]
8	Salt bridges	B: ARG 59 [NH2]	3.21	A: GLU 685 [OE1]
9		B: ARG 59 [NE]	3.21	A: GLU 685 [OE1]
10		B: ARG 56 [NH1]	2.58	A: GLU 696 [OE2]

In summary, our findings suggest that Nsp2 binds to TBK1 without altering TBK1 protein levels or its phosphorylation status. However, the interaction between Nsp2 and TBK1 leads to a decrease in IRF3 phosphorylation, ultimately resulting in reduced IFN-β production.

### Getah virus Nsp2 interrupts the interaction of IRF3 with KPNA3 and KPNA4

Based on the results showing reduced nuclear translocation of IRF3 (Figure 3G), we hypothesised that Nsp2 antagonises IFN-β production by inhibiting the nuclear transport of IRF3. To explore this further, we examined the impact of Nsp2 on the nuclear translocation of IRF3. Karyopherins are molecules that transport cargo across nuclear pore complexes, either into or out of the karyo-compartment (the nucleus), by binding to classic nuclear localisation sequences (cNLS) [29, 30]. It has been reported that nuclear import of IRF3 is mediated by importin α3 (KPNA4) and importin α4 (KPNA3). Additionally, some viruses, including the Japanese encephalitis virus (JEV) and encephalomyocarditis virus (EMCV), have been known to interact with these importins and inhibit the activation of IRF3-responsive promoters [31, 32].

**Figure 5:**
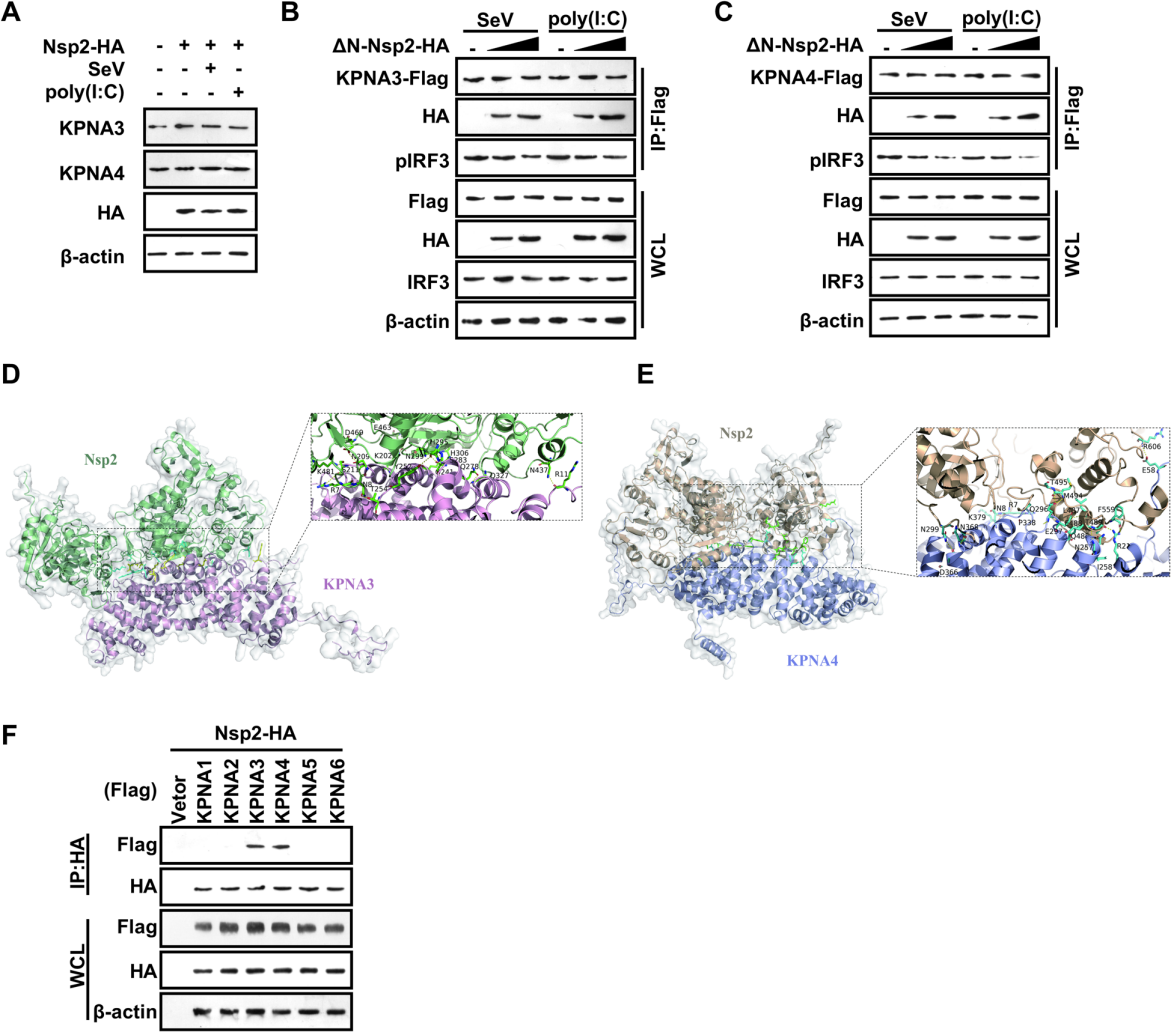
**Getah virus Nsp2 interrupts the interaction of IRF3 with KPNA3 and KPNA4**. **A** PK15 cells were transfected with or without Nsp2 for 24 h, then stimulated with or without poly(I:C) for 12 h. Cells were harvested, and the expression levels of KPNA3 and KPNA4 were analysed by western blotting. **B**, **C** HEK293 T cells were co-transfected with the KPNA3-Flag or KPNA4-Flag construct and ΔN-Nsp2 plasmid (50 ng, 200 ng,) or empty vector. The cells were harvested and lysed at 36 h post-transfection. Immunoprecipitation was performed with Flag antibody, and western blotting was performed with the indicated antibodies **D**, **E** The pale green shade shows the Nsp2, and the light pink shade represents the KPNA3; Nsp2-binding residues are shown in cyan, and KPNA3-binding residues are displayed in yellow. The yellowish colour is the hydrogen bond between Nsp2 and KPNA3 (**D**). The wheat shade represents Nsp2, and the blue and white shades represent KPNA4. The residues involved in Nsp2 binding are marked in green, and those participating in KPNA4 binding are displayed in cyan. A close-up view of the interaction site further emphasises hydrogen bonds, highlighted in yellow (**E**). **F** HEK293 T cells were co-transfected with an HA-tagged Nsp2-expressing plasmid and a Flag-tagged KPNA1-6 6 plasmid or an empty plasmid. At 24 h, Co-immunoprecipitations were performed with whole-cell lysates using the indicated antibodies.

To investigate whether Nsp2 interrupts the nuclear translocation of IRF3 by influencing nuclear transport proteins, we measured the levels of KPNA3 and KPNA4 in cells transfected with either the Nsp2 plasmid or empty vector. The results indicated that Nsp2 expression did not significantly affect the levels of KPNA3 and KPNA4 in PK-15 cells (Figure 5A). Since the N-terminus of Nsp2 influences the level of phosphorylated IRF3 through TBK1, this information may be important for evaluating how Nsp2 affects the nuclear translocation of phosphorylated IRF3. Consequently, we utilised a mutant with an N-terminal deletion of Nsp2 (ΔN-Nsp2, a deletion structure at position 1–155) in the experiments that followed. HEK293 cells were transfected with increasing amounts of the ΔN-Nsp2 plasmid along with either the KPNA3 or KPNA4 plasmid, followed by infection with Sendai virus infection or treatment with poly(I:C).

Co-immunoprecipitations were conducted using anti-KPNA3 and anti-KPNA4 antibodies, followed by western blotting with a p-IRF3 antibody to detect interactions between p-IRF3, KPNA3 and KPNA4. The western blotting results showed that smaller amounts of phosphorylated IRF3 interacted with KPNA3 and KPNA4 in cells that expressed higher levels of ΔN-Nsp2 (Figures 5B, C). This suggests that Nsp2 competitively inhibited the interaction between phosphorylated IRF3 and both KPNA3 and KPNA4, regardless of the N-terminal structure of Nsp2.

We predicted the interactions between Nsp2 and KPNA3 and KPNA4 using the GRAMM Web Server. This analysis confirmed that most of the potential interaction sites are not related to the N-terminal structure of Nsp2. Upon further examination of the interfacial interactions within these complexes, we found that the Nsp2- KPNA3 complex features 10 H-bonds and 6 salt bridges. In comparison, the Nsp2-KPNA4 complex exhibits even more extensive interactions, comprising 14 hydrogen bonds and 8 salt bridges (Figures 5D, E).

The H-bonds are found at the KPNA3 residues ARG 11, along with SER 211, GLN 241, GLN 278, SER 283, LYS 202, ASN 209, TYR 252, and THR 254. The salt bridges occur at residues GLU 242, GLU 275, ASP 282, and LYS 202. For KPNA4, H-bonds are observed at residues ARG 218, ASN 257, GLN 296, ASN 368, GLU 58, GLU 297, PRO 338, ASP 366, and LYS 379. The salt bridges at KPNA4 exist at residues HIS 295, ASP 326, GLU 340, and ASP 366. A detailed list of the amino acid residues involved in this binding is provided in Tables 2 and 3.

**Table 2:** **Hydrogen bonds and salt bridges connected by Nsp2 and KPNA3**

No	Type	Nsp2	Dist.[Å]	KPNA3
1	Hydrogen bonds	ASN4 37 [ND2]	2.52	ARG 11 [O]
2		ARG 7 [NH1]	3.70	SER 211 [O]
3		LYS 481[NZ]	2.12	SER 211 [OG]
4		HIS 306 [N]	3.38	GLN 241 [OE1]
5		GLN 327 [NE2]	2.54	GLN 278 [OE1]
6		ASN 299 [N]	3.73	SER 283 [O]
7		GLU 463 [OE2]	2.95	LYS 202 [NZ]
8		ASP 469 [OD2]	3.89	ASN 209 [ND2]
9		HIS 295 [O]	3.42	TYR 252 [OH]
10		ASN 8 [OD1]	3.40	THR 254 [OG1]
11	Salt bridges	ARG 303 [NE]	2.01	GLU 242 [OE1]
12		ARG 303 [NH1]	3.45	GLU 242 [OE1]
13		ARG 303 [NH2]	3.50	GLU 242 [OE1]
14		LYS 329 [NZ]	3.62	GLU 275 [OE2]
15		LYS 282 [NZ]	2.35	ASP 282 [OD1]
16		GLU 463 [OE2]	2.95	LYS 202 [NZ]

**Table 3:** **Hydrogen bonds and salt bridges connected by Nsp2 and KPNA4**

No	Type	Nsp2	Dist. [Å]	KPNA4
1	Hydrogen bonds	PHE 559 [O]	3.15	ARG 218 [NH1]
2		THR 489 [O]	2.11	ARG 218 [NH2]
3		GLU 488 [O]	3.25	ASN 257 [N]
4		GLU 467 [OE2]	3.71	GLN 296 [N]
5		CYS 301 [O]	3.48	ASN 368 [N]
6		ARG 606 [N]	3.42	GLU 58 [OE2]
7		MET 494 [N]	2.79	GLN 296 [OE1]
8		THR 495 [N]	3.52	GLN 296 [OE1]
9		LEU 487 [N]	3.83	GLU 297 [OE2]
10		GLN 485 [N]	2.58	GLU 297 [OE2]
11		ARG 7 [NH1]	2.20	PRO 338 [O]
12		ASN 299 [ND2]	2.77	ASP 366 [O]
13		HIS 306 [NE2]	3.39	ASP 366 [OD2]
14		ASN 8 [N]	2.84	LYS 379 [O]
15	Salt bridges	GLU 488 [OE1]	2.14	HIS 295 [ND1]
16		GLU 488 [OE1]	3.19	HIS 295 [NE2]
17		ARG 303 [NE]	3.19	ASP 326 [OD1]
18		ARG 303 [NH1]	3.21	ASP 326 [OD1]
19		ARG 303 [NH1]	3.71	ASP 326 [OD2]
20		ARG 7 [NE]	2.68	GLU 340 [OE1]
21		ARG 7 [NE]	3.69	GLU 340 [OE2]
22		HIS 306 [NE2]	3.39	ASP 366 [OD2]

The PDBePISA analysis of the protein–protein interface of the complex indicates that the binding affinities of the docking complexes of Nsp2 with KPNA3 and KPNA4 are −13.2 kcal/mol and −13.3 kcal/mol, respectively. Co-immunoprecipitation experiments demonstrated that Nsp2 selectively interacts with KPNA3 and KPNA4, but not with the other KPNAs (Figure 5F). These results suggest that GETV Nsp2 competitively inhibits the interaction between p-IRF3 and both KPNA3 and KPNA4, thereby inhibiting the expression of IFN-β.

### Overexpression of KPNA3 or KPNA4 restores the transcriptional activity of IRF3 suppressed by GETV Nsp2

**Figure 6:**
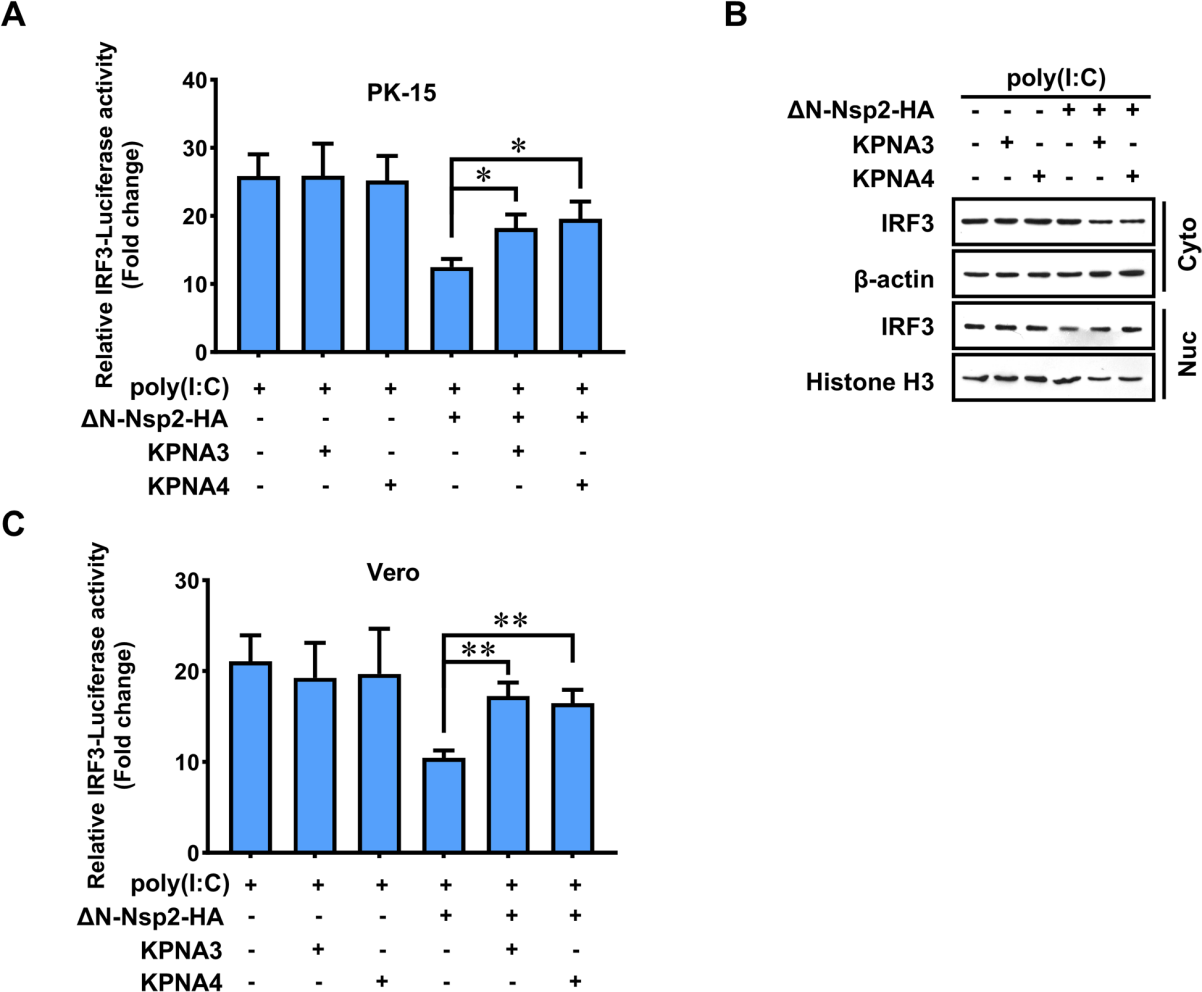
**Overexpression of KPNA3 or KPNA4 recruits IRF3 activity, which is inhibited by GETV Nsp2**. **A** A plasmid expressing ΔN-Nsp2 was co-transfected into PK-15 cells with vector plasmid (–) or a KPNA construct (+) and the IRF3-dependent reporter system. At 24 h post-transfection, cells were stimulated with poly(I·C) for 12 h before a dual-luciferase assay was used to determine IRF3 Activity. Statistical significance of the presented data was determined as follows: * *P* < 0.05. **B** PK-15 cells were transfected or co-transfected with a plasmid encoding GETV ΔN-Nsp2 and a plasmid expressing KPNA3 or KPNA4 for 24 h, then stimulated with poly(I·C) for another 12 h. Protein levels of IRF3 in the cytoplasmic extract and nuclear extract were analysed by western blotting. **C** Vero cells were co-transfected with a plasmid encoding GETV Nsp2 and a KPNA construct and the IRF3-dependent reporter system. At 24 h post-transfection, cells were stimulated with poly(I·C) for 12 h before a dual-luciferase assay was used to determine IRF3 activity. The statistical significance of the presented data was determined as follows: ** *P* < 0.01.

To further demonstrate that Nsp2 inhibits the activation of IRF3 by targeting KPNA3 and KPNA4, we measured IRF3-dependent luciferase activity in cells overexpressing KPNA3 or KPNA4. As anticipated, treatment with poly(I:C) increased the activity of the IRF3-Luc reporter compared to the control. However, cells transfected with ΔN-Nsp2 showed a significant suppression of the IRF3-Luc reporter activity induced by poly(I:C). Notably, the activity of the IRF3-Luc reporter was higher in the groups expressing KPNA3 or KPNA4, compared to those transfected solely with ΔN-Nsp2. This finding indicates that the overexpression of KPNA3 or KPNA4 restored the IRF3 activity that had been reduced by ΔN-Nsp2 (Figure 6A).

Additionally, we analysed protein levels of IRF3 in both the cytoplasm and the nucleus using western blotting. We observed reduced levels of IRF3 in the cytoplasmic fraction and increased levels in the nuclear fraction of cells co-transfected with KPNA3 or KPNA4 and ΔN-Nsp2, compared to cells transfected with ΔN-Nsp2 alone (Figure 6B). Previous studies have shown that GETV Nsp2 interferes with the phosphorylation of STAT1 and its nuclear accumulation, which results in significantly impaired JAK-STAT signalling upon interferon stimulation [33].

Nsp2 may disrupt the downstream feedforward loop of IFN signalling, in addition to competing for importins. To eliminate potential confounding effects, we repeated the IRF3-dependent luciferase activity assay using a Vero cell line known to have deficiencies in IFN expression, and we observed similar results for Vero cells (Figure 6C). Together, these data suggest that GETV Nsp2 inhibits the nuclear localisation of IRF3 by blocking its interactions with importins.

## Discussion

Getah virus (GETV) is a mosquito-borne virus that belongs to the genus Alphavirus in the family Togaviridae. In recent years, it has caused several outbreaks in animals, particularly in southern China, which has drawn significant attention [6, 34]. However, the molecular basis of GETV pathogenicity remains poorly understood. The host’s innate immunity serves as the first line of defence against viral infections, with type I interferons playing a crucial role. Interferons are known for their ability to inhibit virus replication and the resulting pathogenesis by triggering both innate and cell-mediated immune responses.

To survive, almost all viruses have evolved mechanisms to defend themselves against the interferon system. These viral countermeasures target all levels of the IFN system, preventing IFN synthesis and diminishing the IFN response. Through unbiased screening, we identified Nsp2 as a key GETV protein that suppresses IFN-I by targeting IRF3, revealing the underlying mechanisms at play. Our research shed light on the impact of GETV infection on the induction and response of type I interferon, providing new insights into how GETV evades the host’s innate immune system.

Alphaviruses are small, enveloped, positive-sense RNA viruses that belong to the *Togaviridae* family and continue to pose a public health concern. In recent decades, these viruses have reemerged, causing numerous epidemics and outbreaks worldwide [35].

Historically, alphaviruses were categorised as either Old World or New World based on their geographic distribution and the characteristics of the diseases they cause in humans. The New World (NW) alphaviruses include Venezuelan, eastern, and western equine encephalitis viruses (VEEV, EEEV, and WEEV) [35, 36]. Old World alphaviruses include chikungunya virus (CHIKV), Sindbis virus (SINV), o’nyongnyong virus (ONNV), Semliki Forest virus (SFV), Ross River virus (RRV) and Getah virus (GETV).

Various alphaviruses have developed strategies to evade the host's innate antiviral immune response. Chikungunya virus (CHIKV), an arbovirus belonging to the genus Alphavirus, can inhibit the activation of the RIG-I signalling pathway in multiple ways [37–40]. Additionally, the cGAS-STING signalling pathway, which detects endogenous or non-self-DNA in the cytoplasm, has been shown to reduce alphavirus infection [41]. Although alphavirus RNA is not believed to engage cGAS directly, CHIKV infection can cause cellular injury that results in the translocation of nuclear or mitochondrial DNA into the cytoplasm. This translocated DNA can bind to cGAS and stimulate the production of type I interferon [41].

The TF protein of the Sindbis virus (SINV) inhibits the host’s type I interferon responses in a manner that depends on palmitoylation [42]. Research on SINV and the Semliki Forest virus (SFV) has shown that mutations in Nsp2 lead to significant deficiencies in countering the IFN response [23].

Our study confirms the strong antagonistic effect of GETV Nsp2 in inhibiting the production of type I interferons induced by various exogenous stimuli. We found that GETV Nsp2 effectively inhibits the activation of IRF3, which plays a central role in both the RIG-I signalling pathway and the cGAS-STING signalling pathway. By preventing IRF3 activation, GETV Nsp2 hinders the production of type I interferons and pro-inflammatory cytokines, as IRF3 is a crucial transcription factor for their induction. This mechanism could represent a strategic approach by GETV to simultaneously suppress processes in the host's innate immune response processes.

Upon viral infection, activated TBK1 phosphorylates STING at a pLxIS motif, which is also present in MAVS. This phosphorylation creates a docking site for the transcription factor IRF3 [43]. Following this, TBK1 phosphorylates IRF3, which promotes its dimerisation, translocation to the nucleus and, ultimately, its role in regulating de novo gene expression. The phosphorylation of IRF3 occurs in SR regions [43]. This modification is crucial for IRF3’s nuclear translocation and its subsequent binding to the IFN-β promoter. Our study demonstrated that the phosphorylation of IRF3 and its accumulation in the nucleus were reduced in GETV Nsp2-expressing cells. This suggests that phosphorylated IRF3 is responsible for the inhibition of IFN-β production by the Nsp2 of GETV.

Given the close relationship between the phosphorylation of IRF3 and TBK1, we investigated the interaction between Nsp2 and TBK1. Our findings confirmed that Nsp2 can competitively bind to TBK1, resulting in a decrease in the phosphorylation of IRF3, which subsequently leads to reduced production of IFN-β.

TBK1 consists of an N-terminal kinase domain (KD), followed by a ubiquitin-like domain (ULD), and a C-terminal region [44]. The kinase domain of TBK1 has been identified as the crucial region for activating and transducing downstream signalling. Previous studies have shown that autophosphorylation at Ser172 within the kinase domain can activate the TBK1 kinase, which is a key step in virus-triggered signalling [45, 46]. However, our study found that GETV Nsp2 does not bind to the TBK1 KD domain, which aligns with our expectations, as it neither reduces the phosphorylation at Ser172 nor blocks TBK1 activation.

The C-terminal region of TBK1 contains two coiled-coil (CC) regions, with CC2 specifically responsible for interacting with the adaptor proteins TANK (TRAF family member-associated NF-κB activator) and NAP1 (NAK-associated protein 1) [47]. Our research has shown that GETV Nsp2 binds to the CC2 domain of TBK1. Although this binding does not directly affect TBK1 itself, it leads to a reduction in the phosphorylation of IRF3.

From a virological perspective, the non-structural protein polyproteins Nsp1234, Nsp123, and Nsp23 are produced during the replication of GETV. Among these, Nsp2 plays a crucial role due to its essential enzymatic functions, which are necessary for viral replication and protein cleavage. These functions include NTPase, RNA helicase, and RNA triphosphatase.

Nsp2 is composed of 798 amino acids and consists of five structural domains. The N-terminal structural domain includes the N-terminal domain, a stalk, and domain 1B. This is followed by two RecA-like structural domains. The C-terminal region comprises the protease domain and the C-terminal domain, which are connected to the RecA2 domain by a linker.

Our study indicates that the interaction between Nsp2 and TBK1 creates hydrogen bond sites concentrated within the 50–155 amino acids of Nsp2. The segment falls within the N-terminal domain and the stalk region. Recent research has shown that the alphavirus Nsp2 plays a key role in the induction of eEF2 phosphorylation [48]. This process depends on the NTPase activity of Nsp2, which results in elevated cellular cAMP levels. This increase in cAMP subsequently activates eEF2 kinase, leading to the phosphorylation of eEF2 [48]. Notably, the area that interacts with TBK1 corresponds precisely to the NTPase region. It remains to be investigated whether the interaction between Nsp2 and TBK1 influences the biological functions of TRAF or NAP1 in future studies.

The expression of IFN-β changes dynamically in real-time, exhibiting significant differences only at specific time points. In contrast, the expression of the luciferase gene exhibited substantial differences throughout the entire infection stage. This more pronounced expression contributed to the suppression observed in luciferase reporter assays, surpassing the variations noted in transcription data and protein level assays.

Signal transduction pathways activated by dsRNA lead to the activation of IRF3, NF-κB, and AP-1. These factors bind to specific PRD motifs in the IFN-β promoter, mediating the transcriptional induction of type I IFN genes [49]. To evade the antiviral response of host cells, many viruses have developed sophisticated mechanisms to interfere with the signalling pathways of IRF3, NF-κB, and AP-1 [26, 50].

Unlike alphaviruses such as the Chikungunya virus (CHIKV), the Nsp2 protein of GETV exhibits distinct immunomodulatory properties. For instance, CHIKV nsP2 broadly disrupts type I interferon signalling by degrading host transcription factors (e.g., STAT1) or by inhibiting the nuclear translocation of NF-κB [37, 51, 52]. Furthermore, CHIKV nsP2 can significantly antagonise the activation of the IFN-β promoter that is mediated by IRF3- and IRF3/5D [53].

In this study, however, transfection with GETV Nsp2 specifically inhibited the luciferase activity of the PRDIII motif, which has a particular affinity for IRF3. Significantly, it did not affect the luciferase activity of NF-κB and AP-1, which are associated with PRD II and PRD IV, respectively. These findings indicate that GETV Nsp2 interferes explicitly with the IRF3 signalling pathway, rather than with AP-1 or NF-κB. This inhibition disrupts the Sendai Virus-mediated activation of IFN production.

This selective inhibition pattern suggests that GETV has developed a sophisticated immune evasion strategy that operates through dual mechanisms. First, by specifically targeting IRF3, the core transcription factor responsible for the production of type I interferon, the virus dampens the host’s antiviral response while minimizing disruption to the NF-κB/AP-1-mediated inflammatory signalling pathway. This likely helps to maintain partial cellular homeostasis, creating a favourable environment for viral replication.

Secondly, by not broadly suppressing host transcription factors, GETV may reduce apoptosis and extend the window for viral replication. Additionally, the preserved activity of the NF-κB/AP-1 pathways observed in this study may be due to the specific molecular actions of GETV Nsp2. For example, IRF3 activation relies on TBK1/IKKε-mediated phosphorylation and dimerisation, whereas NF-κB activation requires the IKKα/β complex and the subsequent degradation of IκBα.

**Figure 7:**
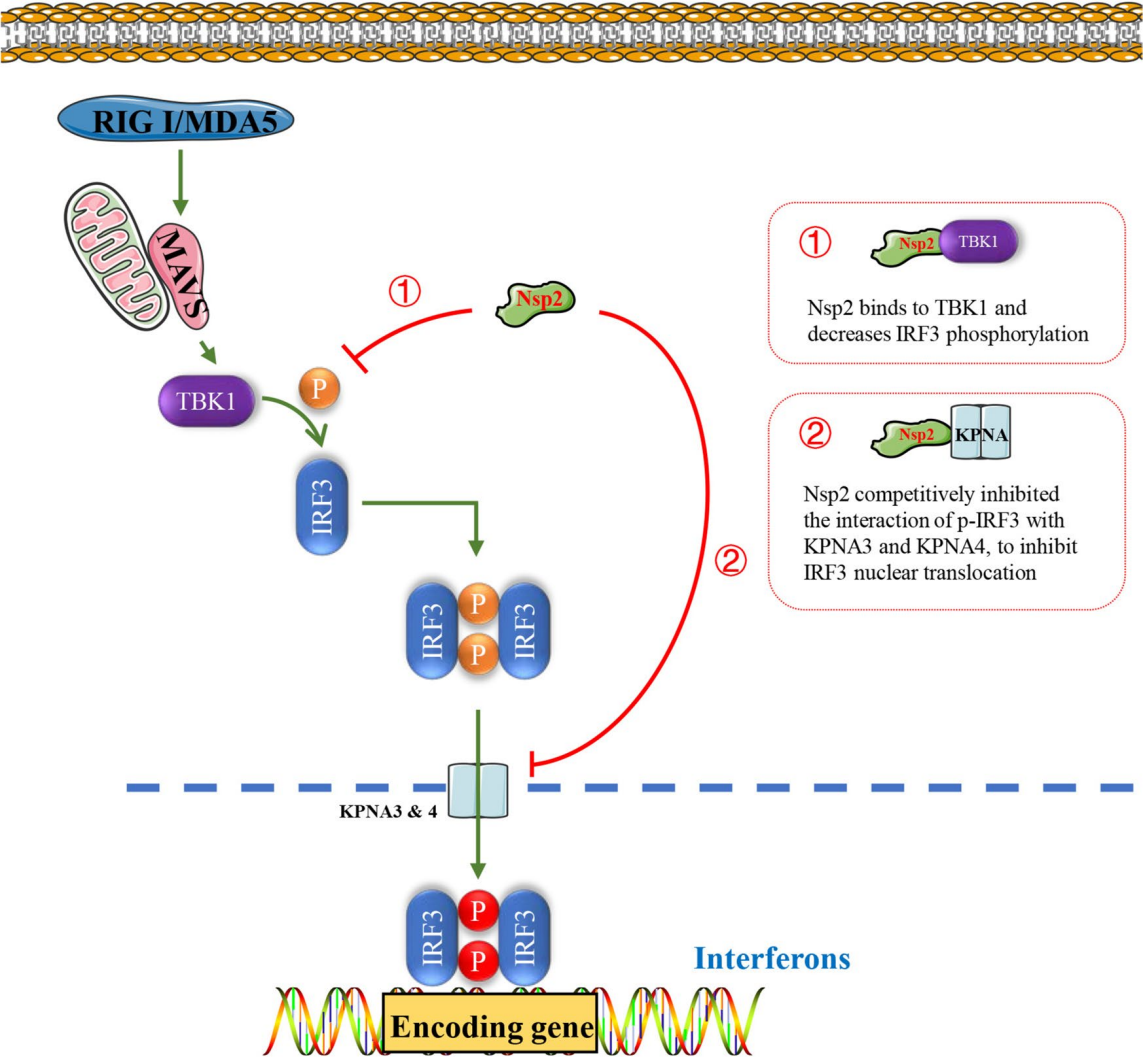
**Schematic diagram of GETV Nsp2 inhibiting the RLR signalling pathway by regulating IRF3 activation**. Nsp2 binds TBK1 to suppress IRF3 phosphorylation. Meanwhile, Nsp2 competitively inhibited the interaction of pIRF3 with KPNA3 and KPNA4, to inhibit IRF3 nuclear translocation.

In summary, we have identified the GETV Nsp2 as a significant inhibitor of host RLR-mediated type I IFN production by interrupting the activation of interferon regulatory factor 3 (Figure 7). However, our study does have some limitations. Currently, our group has completed a DNA-launched infectious clone of the Getah virus.

Moving forward, it is essential to focus on the function of Nsp2 under the condition of GETV infection. Specifically, we need to investigate whether GETV Nsp2 can directly regulate the activity of IRF3 and whether it influences the signalling molecules that connect TBK1 and IRF3. Additionally, it is essential to understand how these biological changes relate to GETV viral replication. Clarifying these questions will enhance our understanding of the molecular mechanism through which GETV Nsp2 inhibits IFN expression. Moreover, our study has provided new insights into how GETV evades the host’s innate immune response.

In summary, the data presented in this study indicate that Nsp2, a crucial viral protein, effectively suppresses the biological functions mediated by IRF3. Specifically, Nsp2 plays a key role in GETV-mediated interference with the RIG-I signalling pathway, which in turn diminishes the host’s innate immune response. This finding highlights Nsp2 as a potential target for antiviral strategies aimed at diseases associated with GETV infection.

## Data Availability

The data generated during this study are available from the corresponding authors upon reasonable request.
